# MicroRNA-9-5p inhibits proliferation and induces apoptosis of human hypertrophic scar fibroblasts through targeting peroxisome proliferator-activated receptor β

**DOI:** 10.1242/bio.051904

**Published:** 2020-12-21

**Authors:** Chi-Yung Chai, I.-Chun Tai, Rui Zhou, Junlong Song, Chaoying Zhang, Shengrong Sun

**Affiliations:** 1 Department of Breast and Thyroid Surgery, Renmin Hospital of Wuhan University, Wuhan, Hubei 430060, China; 2 Reichen Biomedical Co., Ltd., Kaohsiung, Taiwan 81155, ROC; 3 Department of Oncology Surgery, The First Affiliated Hospital of Bengbu Medical College, Bengbu, Anhui 233003, China; 4 School of Basic Medical Sciences, Guangzhou University of Chinese Medicine, Guangzhou, Guangdong 510006, China

**Keywords:** miR-9-5p, PPARβ, Hypertrophic scar, Extracellular matrix, Proliferation, Apoptosis

## Abstract

Hypertrophic scar (HS) is a dermal fibro-proliferative disorder result from abnormal wound healing after skin injury. MicroRNA-9-5p (miR-9-5p) has been reported to be upregulated and closely related to collagen proteins in human dermal fibroblasts. However, the correlation and possible mechanism between miR-9-5p and HS require further investigation. The expressions of miR-9-5p in HS tissues and HS fibroblasts were detected by quantitative real-time PCR (RT-qPCR). The expression level of peroxisome proliferator-activated receptor β (PPARβ) was measured by RT-qPCR assay. The protein levels of PPARβ, α-SMA, Vimentin, COL1A, cyclin D1, bcl-2, and bax were detected by western blot assay. The effect of miR-9-5p and PPARβ on HS fibroblasts proliferation and apoptosis were detected by cell counting kit-8 (CCK-8) and flow cytometry assays. The interaction between miR-9-5p and PPARβ was predicted by TargetScan, and then confirmed by dual-luciferase reporter assay. MiR-9-5p expression was downregulated in HS tissues and HS fibroblasts. MiR-9-5p inhibited the levels of extracellular matrix-associated genes (α-SMA, Vimentin, COL1A) in HS fibroblasts. MiR-9-5p repressed proliferation and induced apoptosis of HS fibroblasts. PPARβ is a target gene of miR-9-5p. The silencing of PPARβ expression hindered proliferation and expedited apoptosis of HS fibroblasts. MiR-9-5p suppressed proliferation and promoted apoptosis of HS fibroblasts by targeting PPARβ. In this paper, we firstly disclosed that miR-9-5p hampered extracellular matrix deposition and proliferation, and induced apoptosis by targeting PPARβ in HS fibroblasts. Our findings provided a new role of miR-9-5p/PPARβ in the occurrence and development of HS fibroblasts, promising a new target for HS.

## Abstract (precis)

**Summary:** Our findings provided a new role of miR-9-5p/PPAR in the occurrence and development of HS fibroblasts, promising a new target for HS.

## INTRODUCTION

Hypertrophic scar (HS) is a dermal fibroproliferative disorder that occurs following deep dermal injury (Ledon et al., 2013). The exceptional proliferation of HS fibroblasts and excessive deposition of extracellular matrix facilitates the formation of HS (Zhu et al., 2013). The HS patient's quality of life, including physically and psychologically, was severely affected by HS due to pruritus, pain, and contractures (Stoddard et al., 2014; Zielins et al., 2014). Despite the great progression in curing HS's therapeutic method, for patients with HS the results of the treatment are still unsatisfactory. Thus, it is imperative to figure out the molecular basis underlying HS for developing more effective therapeutic strategies.

MicroRNAs (miRNAs) identified as a family of short, single-stranded, non-coding RNAs negatively regulate target genes by directly binding to the 3’UTR region of target mRNAs in diverse cellular events (Guo et al., 2010; Treiber et al., 2019). Accumulating evidence indicated that miRNA plays a unique role in numerous diseases, including HS. For example, Zhang et al. reported that miR-130a could promote Akt activation by targeting CYLD, thereby inducing fibroproliferative in HS (2019). Zhou et al. confirmed that miR-519d worked in a suppressive role by blocking proliferation and expediting apoptosis in HS formation (2018). MicroRNA-9-5p (miR-9-5p), a member of the miR-9 family, has been proven to have an anti-fibrotic effect in lung and peritoneal fibrosis (Fierro-Fernández et al., 2015). A recent study reported that miR-9-5p was tightly related to collagen proteins in human dermal fibroblasts (Miguel et al., 2016). Nevertheless, the precise function of miR-9-5p in HS requires further investigation.

Peroxisome proliferator-activated receptor β (PPARβ), a subtype of ligand-inducible nuclear receptors, has emerged as an important multifaceted role in skin wound healing (Montagner et al., 2015; Neels and Grimaldi, 2014). Some studies have shown that knockdown of PPARβ could elevate epidermal hyperplasia and inflammation, and suggest a potential therapeutic target for dermal fibrosis in mice (Sng et al., 2018). Notably, a recent reported that confirmed that PPARβ is upregulated in hypertrophic scar tissues, and its overexpression boosted proliferation and cell movement ability in human HS fibroblast cells, implying the important role of PPARβ in HS formation (Xiao et al., 2015). Nevertheless, the underlying regulatory mechanism of PPARβ in HS formation is still unclear.

In this paper, we verified the impact of miR-9-5p and PPARβ in HS fibroblasts and figured out the interaction between miR-9-5p and PPARβ, providing evidence for the miR-9-5p/PPARβ axis in suppressing the HS formation and development.

## RESULTS

### MiR-9-5p is downregulated in HS tissues and fibroblasts

**Fig. 1.**
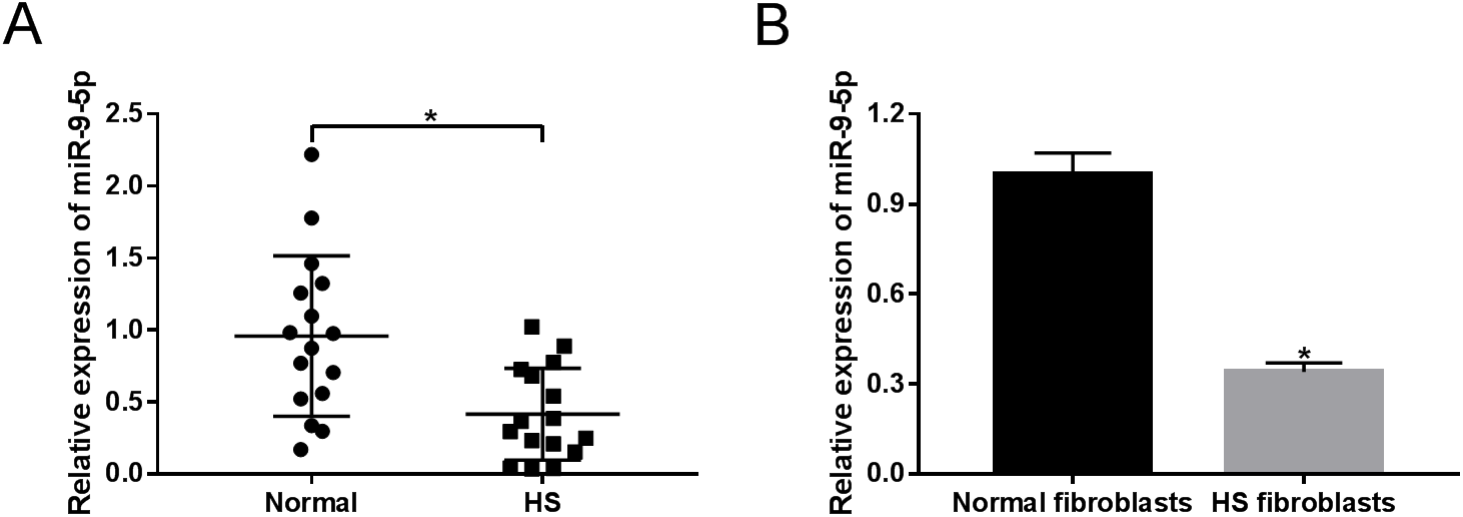
**Expression patterns of miR-9-5p in HS tissues and fibroblasts.** (A) RT-qPCR assay was carried out to detect the expression of miR-9-5p in 16 pairs of HS tissues and adjacent normal tissues. (B) Expression of miR-9-5p in HS fibroblasts and normal fibroblasts was assessed by RT-qPCR. *P<0.05.

To explore the functional role of miR-9-5p in HS, its expression patterns were initially measured by RT-qPCR assays. As shown in Fig. 1A, miR-9-5p expression was markedly decreased in HS tissues relative to adjacent normal tissues. Then, we further proved that miR-9-5p was lower expressed in HS fibroblasts in contrast to normal fibroblasts (Fig. 1B). These results suggested that abnormal expression of miR-9-5p might be implicated in the pathogenesis of HS.

### MiR-9-5p inhibits the expression of extracellular matrix-associated genes in HS fibroblasts

**Fig. 2.**
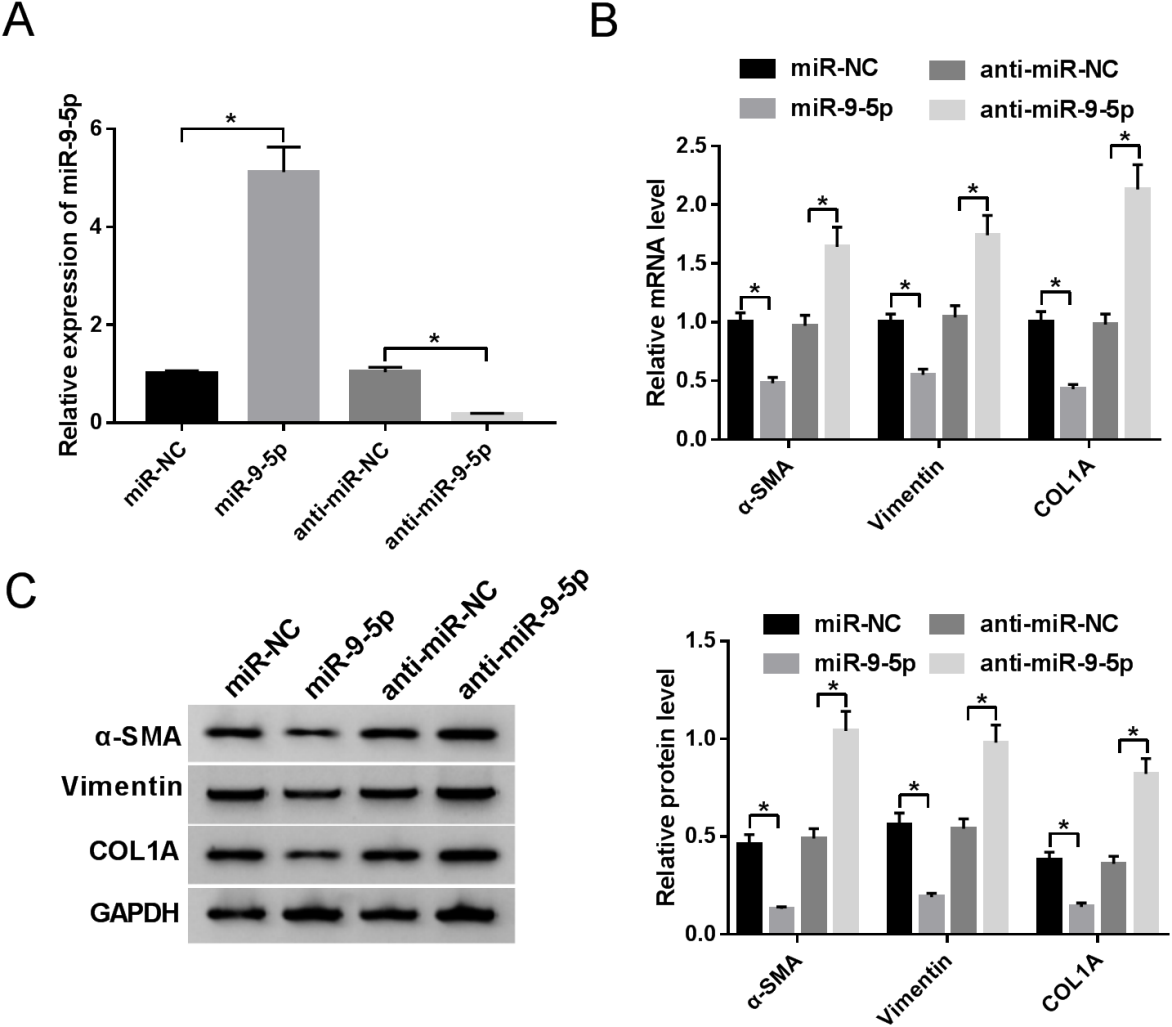
**MiR-9-5p inhibits the expression of extracellular matrix-associated genes in HS fibroblasts.** (A) Transfection efficiency of pcDNA3.1-miR-9-5p or anti-miR-9-5p in HS fibroblasts. (B) RT-qPCR analysis of α-SMA, Vimentin, and COL1A expression levels in transfected HS fibroblasts. (C) Western blot analysis of α-SMA, Vimentin, and COL1A expression levels in transfected HS fibroblasts. *P<0.05.

**Fig. 3.**
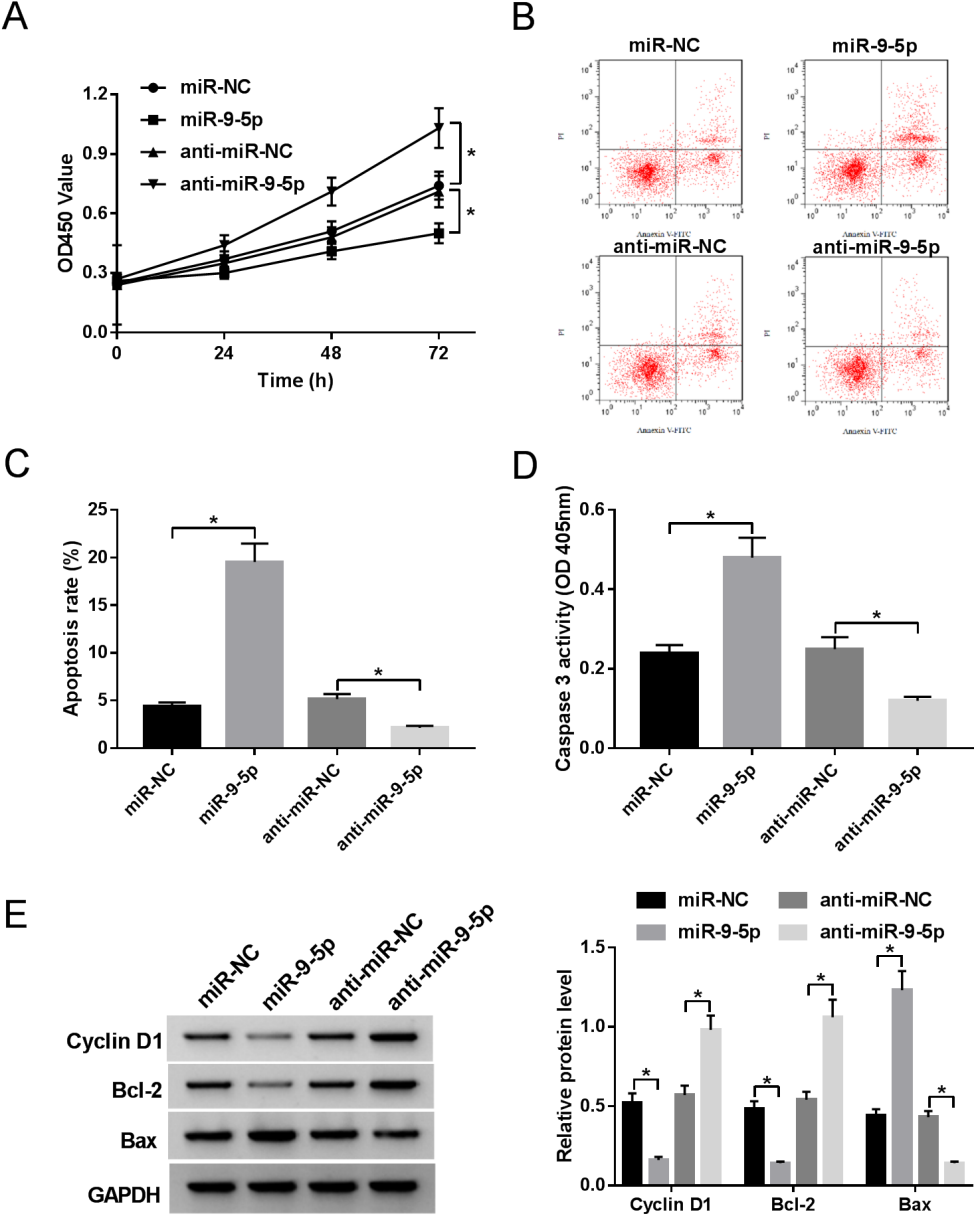
**MiR-9-5p inhibits proliferation and induces apoptosis of HS fibroblasts.** (A) CCK-8 assay was performed to determine the proliferative ability of HS fibroblasts transfected with pcDNA3.1-miR-9-5p or anti-miR-9-5p. (B,C) Apoptosis rates in transfected HS fibroblasts were detected by flow cytometry assay. (D) Caspase-3 activity analysis in transfected HS fibroblasts. (E) Expression levels of cyclin D1, bcl-2, and bax were measured by western blot assay. *P<0.05.

Next, to investigate the functional role of miR-9-5p in HS fibroblasts, the overexpression vector and knockdown antisense RNA (anti-RNA) of miR-9-5p were synthesized. According to the result shown in Fig. 2A, the expression level of miR-9-5p was upregulated in pcDNA3.1-miR-9-5p-transfected HS fibroblasts, and the miR-9-5p expression level was downregulated in anti-miR-9-5p-transfected HS fibroblasts, when compared with respective control groups. Subsequently, we used the overexpression or knockdown systems to identify the effect of miR-9-5p on the extracellular matrix. The results indicated that α-SMA, Vimentin, and COL1A mRNA level were evidently reduced after the introduction with miR-9-5p mimic, while these extracellular matrix-associated genes mRNA levels were prominently improved after downregulating the expression of miR-9-5p (Fig. 2B). Moreover, western blot demonstrated that overexpression of miR-9-5p effectively hindered α-SMA, Vimentin, and COL1A proteins level of HS fibroblasts, and, conversely, deficiency of miR-9-5p led to an overt increase in these proteins level (Fig. 3B). In short, miR-9-5p could suppress the accumulation of extracellular matrix in HS fibroblasts.

### MiR-9-5p inhibits proliferation and induces apoptosis of HS fibroblasts

To observe the effect of miR-9-5p on the proliferation and apoptosis of HS fibroblasts, cell counting kit-8 (CCK-8) and flow cytometry assays were executed. The results of CCK-8 assays suggested that upregulation of miR-9-5p retarded proliferative ability of HS fibroblasts, whereas downregulation of miR-9-5p facilitated the proliferation of HS fibroblasts (Fig. 3A). Furthermore, elevated cell apoptosis and the increased expression of caspase-3 were observed owing to overexpression of miR-9-5p, however, the silencing of miR-9-5p produced the opposite results (Fig. 3B–D). To further verify the function of miR-9-5p in proliferation and apoptosis of HS fibroblasts, cyclin D1, bcl-2, and bax expression levels were assessed. Western blot results demonstrated that miR-9-5p caused a marked decrease of cyclin D1 and bcl-2 expression, or an evident increase of bax expression in HS fibroblasts, while deficiency of miR-9-5p elicited contrary results (Fig. 3E). Taken together, these data revealed that miR-9-5p could repress proliferation and promote apoptosis of HS fibroblasts.

### *PPARβ* is a target gene of miR-9-5p

**Fig. 4.**
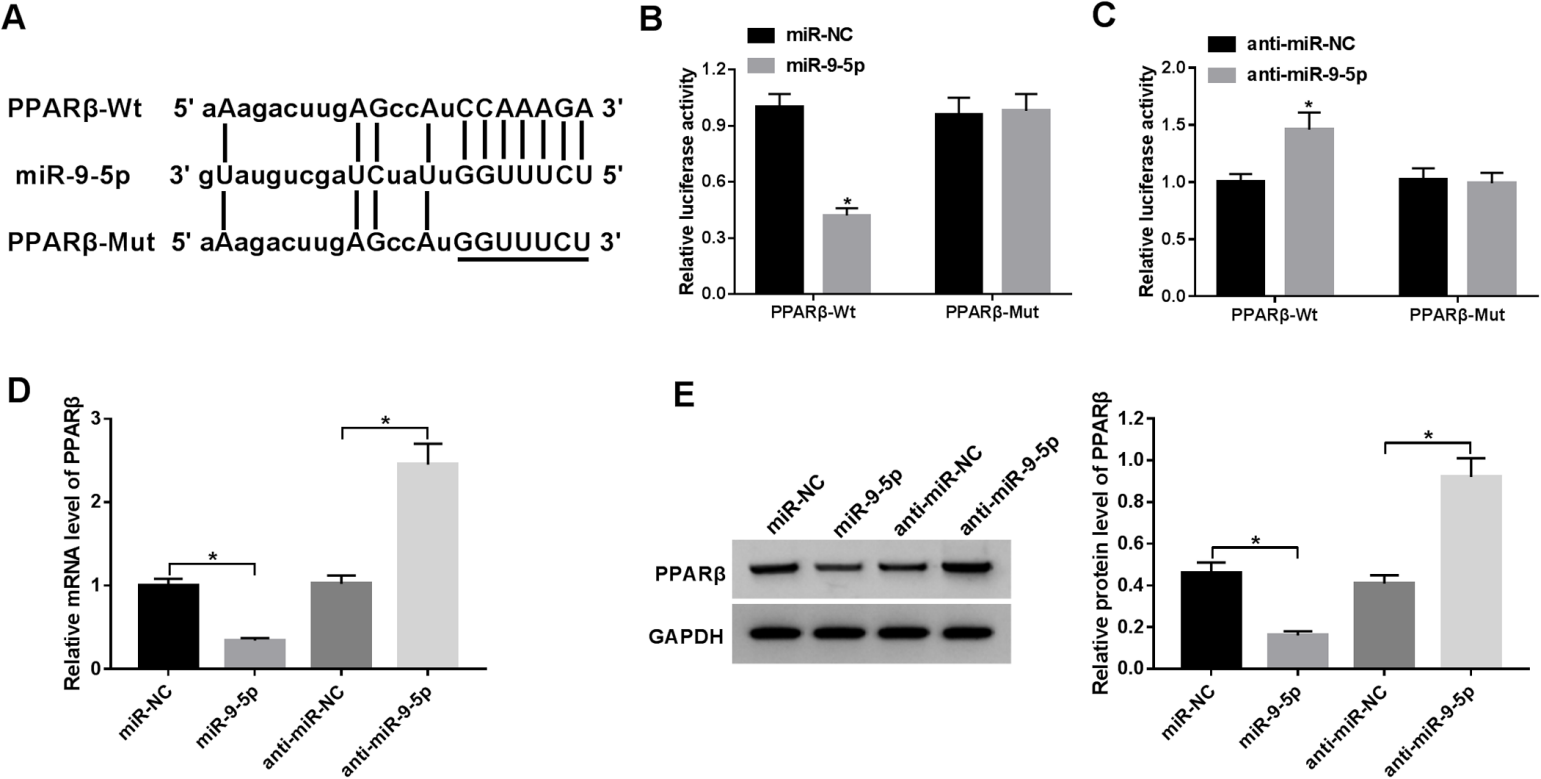
***PPARβ* is a target gene of miR-9-5p.** (A) The binding sites between miR-9-5p and PPARβ and the sequences of PPARβ-Mut. (B,C) The interactions between miR-9-5p and PPARβ were testified by luciferase activity analysis. (D) RT-qPCR analysis of PPARβ in HS fibroblasts transfected with pcDNA3.1-miR-9-5p or anti-miR-9-5p. (E) Western blot analysis of PPARβ in transfected HS fibroblasts. *P<0.05.

It has widely accepted that miRNAs can exert the function by interacting with the target gene expressions. Thus, TargetScan online software (www.targetscan.org) was used to search for the potential target of miR-9-5p. As a result, miR-9-5p harbored some complementary binding sites to 3′UTR of PPARβ (Fig. 4A). To further validate the direct binding between miR-9-5p and PPARβ, WT-PPARβ and MUT-PPARβ reporters were co-transfected with miR-NC, miR-9-5p, anti-NC, or anti-miR-9-5p into HS fibroblasts and then luciferase assay was performed. The results exhibited that ectopic expression of miR-9-5p constrained the luciferase activity of PPARβ-Wt reporter, and miR-9-5p depletion intensified luciferase activity of PPARβ-Mut reporter. Nevertheless, the upregulation or downregulation of miR-9-5p had no effect on these luciferase activities (Fig. 4B,C). Also, RT-qPCR and western blot results disclosed that manipulation of miR-9-5p expression could change the expression of PPARβ, presenting that both mRNA and protein levels of PPARβ were reduced in HS fibroblasts transfected with miR-9-5p and were increased by the knockdown of miR-9-5 (Fig. 4D,E). Collectively, miR-9-5p interacts with PPARβ to block its expression.

### Silencing of PPARβ expression inhibits proliferation and induces apoptosis of HS fibroblasts

**Fig. 5.**
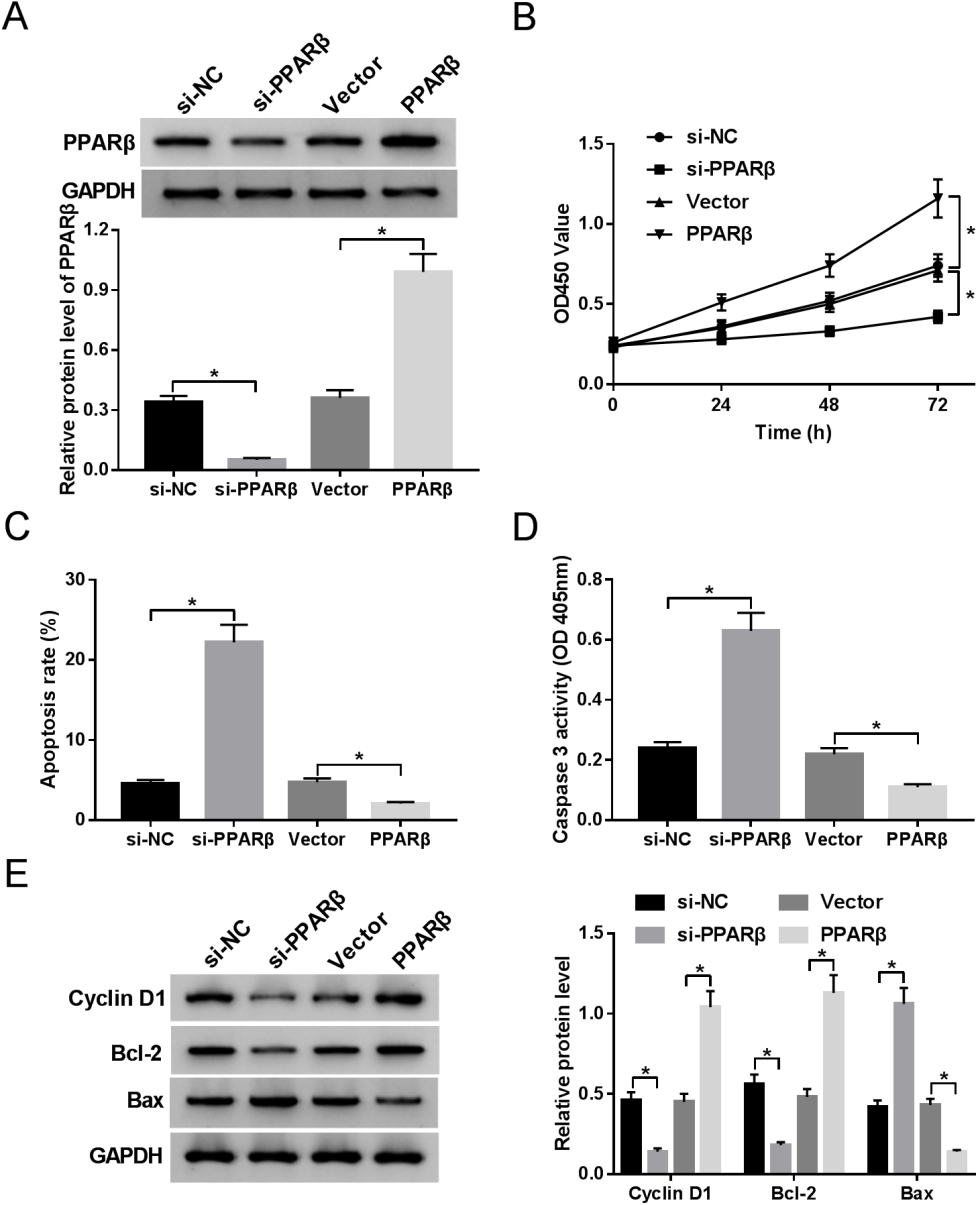
**Silencing of PPARβ expression inhibits proliferation and induces apoptosis of HS fibroblasts.** (A) Transfection efficiency of si-PPARβ or PPARβ in HS fibroblasts. (B) Proliferation analysis in si-PPARβ or PPARβ-transfected HS fibroblasts. (C) Apoptosis rates in transfected HS fibroblasts were measured by flow cytometry assay. (D) Caspase-3 activity analysis in transfected HS fibroblasts. (E) Cyclin D1, bcl-2, and bax expression levels in transfected HS fibroblasts were detected by western blot assay. *P<0.05.

Then, to probe the role of PPARβ in HS fibroblasts, knockdown small interference RNA (siRNA) and overexpression of PPARβ were synthesized. RT-qPCR results showed that the expression of PPARβ was distinctly decreased in HS fibroblasts transfected with si-PPARβ in respect to cells transfected with si-NC, whereas the PPARβ expression level was upregulated in PPARβ-transfected HS fibroblasts versus cells transfected with an empty vector (Fig. 5A). Afterward, the knockdown and overexpression systems were further used to explore the function of PPARβ, including proliferation and apoptosis. The results displayed that HS fibroblast proliferation was prominently impeded after silencing expression of PPARβ and significantly elevated after the transfection of PPARβ (Fig. 5B). Whereafter, we further investigated the influence of PPARβ on the apoptosis of HS fibroblasts. A flow cytometry assay manifested that the knockdown of PPARβ strikingly induced cell apoptosis and the overexpression of PPARβ dramatically hindered the apoptosis of HS fibroblasts (Fig. 5C). Similar to the flow cytometry results, the silence of PPARβ by si-PPARβ triggered an apparent reinforcement in caspase-3 activity, while instruction of PPARβ resulted in an evident decline in caspase-3 activity in HS fibroblasts (Fig. 5D). In addition, western blot results confirmed that PPARβ downregulation led to the decrease of cyclin D1 and bcl-2 expression, and the improvement of bax expression in HS fibroblasts, whereas the overexpression of PPARβ produced the opposite results (Fig. 5E). All these data indicate that PPARβ could induce proliferation and inhibit apoptosis of HS fibroblasts.

### MiR-9-5p inhibits proliferation and induces apoptosis of HS fibroblasts by targeting *PPARβ*

**Fig. 6.**
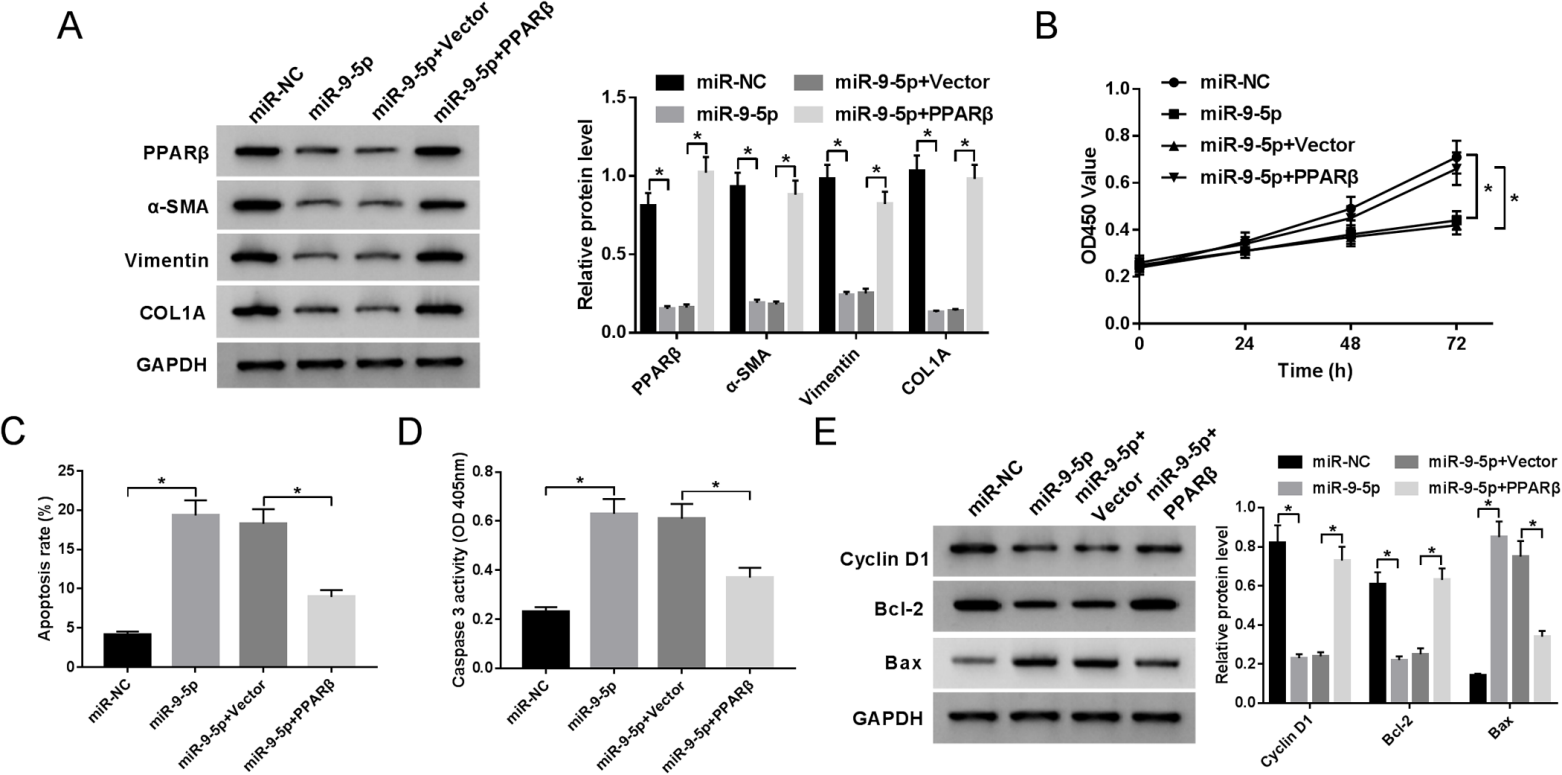
**MiR-9-5p inhibits proliferation and induces apoptosis of HS fibroblasts by targeting PPARβ.** (A) Western blot analysis of PPARβ, α-SMA, Vimentin, and COL1A expression levels in HS fibroblasts transfected with miR-NC, miR-9-5p, miR-9-5p+Vector or miR-9-5p+PPARβ. (B) Proliferation analysis in transfected HS fibroblasts. (C,D) Flow cytometry analysis of apoptosis and caspase-3 activity analysis in transfected HS fibroblasts. (E) Cyclin D1, bcl-2, and bax expression levels in transfected HS fibroblasts were assessed by western blot assay. *P<0.05.

As mentioned above, we inferred that miR-9-5p exerts its function by targeting *PPARβ* in HS fibroblasts. In order to validate the assumption, rescue experiments were implemented by introducing miR-NC, miR-9-5p, miR-9-5p+Vector or miR-9-5p+PPARβ. As displayed in Fig. 6A, miR-9-5p curbed the protein level of PPARβ, α-SMA, Vimentin, and COL1A, which was effectively eliminated by regaining of PPARβ, suggesting that miR-9-5p could repress the accumulation of extracellular matrix partially by modulating PPARβ. Afterward, cell proliferation and apoptosis were also assessed. CCK-8 results confirmed that the upregulation of miR-9-5p conspicuously retarded proliferation, while restoration of *PPARβ* expression greatly attenuated the effect (Fig. 6B). Then, flow cytometry of apoptosis and caspase-3 activity analysis indicated that the re-introduction of PPARβ substantially overturned miR-9-5p-triggered enhancement in apoptotic rate and caspase-3 activity in HS fibroblasts (Fig. 6C,D). Additionally, overexpression of miR-9-5p reduced the expression levels of cyclin D1 and bcl-2, and accelerated bax expression level, while recovery of PPARβ expression markedly attenuated these effects (Fig. 6E). All these results suggested that miR-9-5p hampered proliferation and expedited apoptosis via targeting *PPARβ* in HS fibroblasts.

## DISCUSSION

Increasing evidence has shown that miRNAs play a crucial regulatory role in the occurrence and development of HS (Guo et al., 2019; Liu et al., 2018; Zhou et al., 2015). It has been reported that dysregulation of miRNA is implicated with extracellular matrix production, proliferation, trans-differentiation, and apoptosis in HS fibroblasts (Bi et al., 2017; Liu et al., 2019, 2016; Qi et al., 2019). Remarkably, the excessive proliferation of fibroblasts and the excessive accumulation of extracellular matrix are the primary reason for HS (Aarabi et al., 2007). To our knowledge, the exact function of miRNAs in HS is still unclear. Therefore, in the present study, miR-9-5p as a new regulatory factor in HS fibroblasts was investigated. Moreover, a prior study presented that miR-9-5p worked in a suppressive role in fibrogenesis of skin fibrosis (Miguel et al., 2016).

In this research, we first found that miR-9-5p was lower expressed in HS tissues and HS fibroblasts when compared with their respective control groups. Our study also found that the overexpression of miR-9-5p hindered extracellular matrix-related genes (α-SMA, Vimentin, and COL1A) expression levels in HS fibroblasts. α-SMA and Vimentin are considered the most cytoskeletal proteins (Gonlusen et al., 2001), COL1A is associated with the fibril-forming type I collagen (Meng et al., 2019). Thus, the upregulation of α-SMA, Vimentin, and COL1A implied the accumulation of extracellular matrix, thereby promoting the fibril-forming of HS fibroblasts. That is to say, miR-9-5p served as an inhibitor in the formation of HS fibroblasts. Furthermore, miR-9-5p upregulation blocked proliferation and induced apoptosis of HS fibroblasts, suggesting that miR-9-5p plays a pivotal role in the development of HS fibroblasts.

In recent years, it has been widely accepted that miRNA could exert the function via the interaction of mRNAs (Cloonan, 2015; Wu et al., 2018). Hence, to explore the molecular mechanism of miR-9-5p in HS fibroblasts, we further search the potential target genes of miR-9-5p. Above all, bioinformatics analysis showed that miR-9-5p contained some binding sequences with PPARβ, and then, the predicted relationship was confirmed with a luciferase reporter assay. Simultaneously, study found that the expression level of *PPARβ* was negatively associated with miR-9-5p expression level in HS fibroblasts. In other words, miR-9-5p interacted with *PPARβ* expression to repress its expression. *PPARβ*, an important member of the nuclear receptor supergene family, is identified as having an accelerative role in the proliferation and movement of human HS fibroblasts (Xiao et al., 2015).

Moreover, previous studies have shown that *PPARβ* played the anti-apoptotic role in keratinocytes by regulating the Akt1 signaling pathway (Di-Poï et al., 2002). To investigate whether *PPARβ* has the same function in HS fibroblasts, *PPARβ* gain-of-function or lack-of-function was obtained. Data displayed that silence of *PPARβ* by si-PPARβ impeded proliferation and contributed to apoptosis in HS fibroblasts, while the overexpression of PPARβ got the opposite results. Meanwhile, the enhanced levels of cyclin D1 and bcl-2, and reduced bax level further verified these results. The main function of Cyclin D1 is to promote cell proliferation. Bcl-2 was an anti-apoptotic factor and Bax was a pro-apoptotic factor. Therefore, cyclin D1 and Bcl-2 increased, and bax decreased, which promoted proliferation and inhibited apoptosis. In all, we verified that PPARβ could work as a promoting factor in HS fibroblasts development.

Based on the above findings, we inferred that miR-9-5p could regulate HS fibroblasts’ formation and development by targeting *PPARβ*. To verify this hypothesis, the remedial experiment was implemented. Not surprisingly, re-introduction of PPARβ abrogated miR-9-5p-stimulated reduction in α-SMA, Vimentin, and COL1A protein levels. Moreover, α-SMA and Vimentin are considered the most cytoskeletal proteins, COL1A is associated with the fibril-forming type I collagen. The upregulation of a-SMA, vimentin, and COL1A increased the excessive deposition of extracellular matrix, thereby promoting the fibril-forming of HS fibroblasts. Similarly, miR-9-5p upregulation retarded proliferation and boosted apoptosis, whereas these effects were mitigated after co-transfection with PPARβ.

In conclusion, our study disclosed that miR-9-5p hampered extracellular matrix deposition and proliferation, and induced apoptosis by targeting PPARβ in HS fibroblasts. Our findings provided a new strategy for hindering HS occurrence and development.

## MATERIALS AND METHODS

### Clinical specimens and fibroblast culture

Sample of the HS tissues and paired normal skin tissues were obtained from 16 HS patients who underwent scar surgery at Renmin Hospital of Wuhan University. Every participant signed written informed consent. This study was approved by the Ethics Committee of Renmin Hospital of Wuhan University, China.

Human fibroblasts were collected from the American Type Culture Collection (ATCC, Manassas, VA, USA) and were maintained in an incubator with an atmosphere containing 5% CO_2_ at 37°C in Dulbecco's modified Eagle's medium (DMEM, Thermo Fisher Scientific, Carlsbad, CA, USA) containing 10% fetal bovine serum (FBS, Thermo Fisher Scientific).

### Cell transfection

*PPARβ* siRNAs (si-PPARβ) and scrambled negative control (si-NC) were obtained from Genechem (Shanghai, China). For the overexpression of *PPARβ* (ACCESSION: NM_001171818; Forward: GCTCTAGAGCGGAGCGTGTGACGCTGCG, Reverse: GGGGTACCTTAAATATTTAATTCCCATT), the full-length sequences of *PPARβ* were PCR-amplified and sub-cloned into the pcDNA3.1 empty vector (Invitrogen, Carlsbad, CA, USA). miR-9-5p mimic (miR-9-5p), corresponding scrambled negative control (miR-NC), miR-9-5p inhibitor (anti-miR-9-5p) or scrambled negative control (anti-miR-NC) was purchased from GenePharma (Suzhou, China). All oligonucleotides (40 nM) and plasmids (2 μg) were transfected into HS fibroblasts using Lipofectamine 2000 reagents (Invitrogen) referring to the operation manual.

### RNA extraction and quantitative real-time PCR (RT-qPCR)

Total RNA was isolated from HS tissues and HS fibroblasts using TRIzol reagent (Invitrogen). Extracted RNA (miR-9-5p and *PPARβ*) was synthesised with complementary DNA (cDNA) by using miScript Reverse Transcription (RT) reagent (Qiagen, Dusseldorf, Germany) or PrimeScript RT reagent (Takara, Shiga, Japan), respectively. The cDNA amplification of miR-9-5p and PPARβ was carried out with an SYBR Green PCR kit (Takara) on the Thermal Cycler system (CFX-6, Bio-Rad, California, USA). The 2^−ΔΔCt^ method was used to calculate the relative expression levels of miR-9-5p and *PPARβ*. U6 small nuclear RNA (snRNA) worked as an endogenous control to the normalization of miR-9-5p expression, glyceraldehyde-3-phosphate dehydrogenase (GAPDH) acted as an internal reference to normalization of PPARβ expression. The primer sequences were presented in Table 1.

**Table 1.**
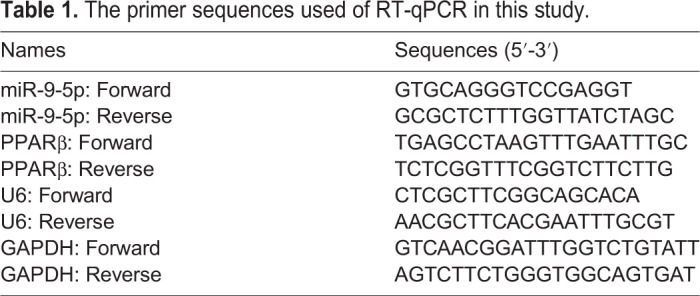
The primer sequences used of RT-qPCR in this study.

### Western blot assay

The protein expressions of PPARβ, α-SMA, Vimentin, COL1A, cyclin D1, bcl-2, and bax in HS fibroblasts with various treatments were detected with western blot assay based on the manufacturer's instructions. Firstly, HS fibroblasts were lysed by pre-cold RIPA buffer (Thermo Fisher Scientific, Waltham, MA, USA) containing protease inhibitors. Extracted proteins (40 μg) were loaded onto 10% sodium dodecyl sulfate-polyacrylamide gel electrophoresis (SDS-PAGE) and then transferred onto a polyvinylidene fluoride (PVDF) membrane (Millipore, Bedford, MA, USA). The membrane was sealed with 5% non-fat milk for 1 h at room temperature, followed by incubation with primary antibody against, PPARβ (1:500, sc-74517, Santa Cruz Biotechnology, Santa Cruz, CA, USA), α-SMA (1:1000, ab7817, Abcam, Cambridge, UK), Vimentin (1:1000, ab92547, Abcam) and collagen type 1 α1 (COL1A; 1:1500, ab6308, Abcam), cyclin D1 (1:1000, ab134175, Abcam), bcl-2 (1:1000, ab32124, Abcam), bax (1:1000, ab69643, Abcam), and GAPDH (1:1000, ab8245, Abcam) at 4°C overnight. The membranes and horseradish peroxidase (HRP)-conjugated secondary antibody were incubated at 37°C for 2 h. The protein bands were detected with an ECL detection kit (Thermo Fisher Scientific).

### Cell proliferation assay

Cell proliferation was monitored by CCK-8, (Sigma-Aldrich, St. Louis, MO, USA) assay. Transfected fibroblasts (6×10^3^ cells per well) were seeded into 96-well plates for 24 h at 37°C, and then cells were added with 10 µl CCK-8 solution (Sigma-Aldrich) and incubated for 2 h. Finally, the absorbance values were measured at different time points (0, 24, 48, and 72 h) under a microplate reader at 450 nm.

### Cell apoptosis and caspase 3 activity assay

Fibroblasts apoptosis was detected with flow cytometry and caspase 3 activity assay at 48 h after transfection. Briefly, for flow cytometry assay, transfected fibroblasts were harvested and washed with PBS (Invitrogen). Fibroblasts were resuspended with binding buffer and stained with 5 μl Annexin (V-fluorescein isothiocyanate) V-FITC and 10 μl Propidium Iodide (PI) according to the manufacturers’ instructions. Apoptotic fibroblasts were detected using FACSCalibur (BD Bioscience, San Jose, CA, USA). For caspase 3 activity assay, fibroblasts were lysed and incubated with caspase 3 substrate (Ac-DEVD-pNA, 2 mM) in reaction buffer at 37°C for 2 h. Finally, the absorbance values at 405 nm were assessed with the microplate reader (BioTek Instruments, Inc.,).

### Dual-luciferase reporter assay

According to the bioinformatics prediction results, luciferase reporter assay was carried out. In this assay, partial sequences of PPARβ 3′UTR possessing wild-type or mutant-type *PPARβ* (WT; forward: AGCTTTGTTTAAAC CCTTTCTCTCTCCACCCCCC, reverse: GCTCTAGA GCTGGAGCAGGATCAGTTGG, PPARβ MUT; forward: AGCCATGGUUUCUAACACTAAGCTCTCT, reverse: AGTGTTAGAAACCATGGCTCAAGTCTTT) miR-9-5p targeting site were synthesized and inserted into luciferase reporter pmirGLO vector (Promega, Madison, WI, USA). And then, HS fibroblasts were co-transfected with the constructed plasmids (PPARβ WT or PPARβ MUT) and miR-9-5p or anti-miR-9-5p. At 24 h post-transfection at 37°C, luciferase activities of fibroblasts were analyzed with the dual luciferase reporter assay kit (Promega).

### Statistical analysis

Data were presented as the mean±standard deviation (s.d.). SPSS 20.0 software (IBM, Chicago, IL, USA) was used to execute statistical analysis. Statistical comparisons containing two groups or more groups were studies with Student’s *t*-test or one-way analysis of variance (ANOVA). Each experiment was repeated three times. *P*<0.05 was considered statistically significant.
